# Affinity-Driven Transfer Learning for Load Forecasting

**DOI:** 10.3390/s24175802

**Published:** 2024-09-06

**Authors:** Ahmed Rebei, Manar Amayri, Nizar Bouguila

**Affiliations:** Concordia Institute for Information Systems Engineering, Montreal, QC H3G1M8, Canada; ahmed.rebei@concordia.ca (A.R.); manar.amayri@concordia.ca (M.A.)

**Keywords:** task affinity score, transfer learning, load forecasting

## Abstract

In this study, we introduce an innovative method for load forecasting that capitalizes on the concept of task affinity score to measure the similarity between various tasks. The task affinity score emerges as a superior technique for assessing task similarity within the realm of transfer learning. Through empirical evaluation on a synthetic dataset, we establish the superiority of the task affinity score over traditional metrics in task selection scenarios. To operationalize this method, we unveil the Affinity-Driven Transfer Learning (ADTL) algorithm to enhance load forecasting precision. The ADTL algorithm enriches the transfer learning framework by incorporating insights from both pre-trained models and datasets, thereby augmenting the accuracy of load forecasting for new and unseen datasets. The robustness of the ADTL algorithm is further evidenced through its application to two empirical datasets, namely the dataset provided by the Australian Energy Market Operator (AEMO) and the Smart Australian dataset. In conclusion, our research underscores the important role of the task affinity score in refining transfer learning methodologies for load forecasting applications.

## 1. Introduction

Load forecasting is the process of predicting future electricity demand. It is an essential task for electricity grids and power system operators to allow for proper functioning and maintain a reliable supply of electricity. Therefore, accurate forecasting is imperative to plan and manage electricity generation, transmission, storage, and distribution. In addition, it is crucial to ensure the stability and reliability of power grids and make informed decisions about electricity generation and transmission capacity [1,2,3]. In recent years, the electricity demand has been increasing due to the emergence of new technologies. The complexity and variability of electricity data are increasing, making it more challenging to address the load forecasting problem.

Many factors can impact the accuracy of load forecasting. For example, weather patterns, economic conditions, changes in electricity consumption patterns, extreme weather events such as heatwaves or cold spells, changes in economic activity, and the adoption of energy-efficient technologies can significantly alter electricity demand. Additionally, the consumption patterns of individual customers can vary considerably over time, making it difficult to predict the overall load on the grid accurately.

Various statistical algorithms have been developed for load forecasting to address these challenges. Before deep learning models became popular, several statistical methods were used to predict future values, such as the autoregressive integrated moving average (ARIMA) [4] and exponential smoothing [5]. However, these methods assume that the data are stationary, which is not valid for electricity demand. Electricity demand is often volatile and exhibits complex trends and seasonal patterns that traditional statistical models cannot capture. Therefore, many machine learning methods have been proposed to mitigate this issue [6], such as support vector regressors [7], fuzzy logic [8], artificial neural networks (ANNs) [9,10,11], radial basis functional networks (RBFNs) [12], and hybrid methods [13,14,15]. In recent years, neural networks, especially recurrent neural networks (RNNs), have become famous for forecasting because they can model nonlinear features and take into account the temporal structure of the data, making them more effective at capturing the evolution of load data [16,17]. More complex approaches have also been proposed in the literature, combining different types of neural networks. In [18], for example, the authors proposed a new forecasting approach that considers both temporal and spatial features using a graph convolution network (GCN) and a multiresolution convolutional neural network (CNN) for short-term wind power forecasting. Similarly, Jiang [19] focused on developing a new learning mechanism to enhance the mapping capability of multi-step demand in building energy forecasting. He also proposed a deep-chain echo state network (DCESN) to effectively prevent error accumulation compared to sliding-window echo state networks and LSTM models.

In addition to the trivial forecasting problem, the context of load forecasting presents some other issues—mainly data scarcity and source volatility. One promising approach for addressing the load forecasting problem is transfer learning. This machine learning technique allows a model to quickly adapt to new tasks by learning from past experiences. Transfer learning has been successfully applied to a variety of functions in various fields, including computer vision, natural language processing, and robotics [20,21,22,23,24]. In the context of load forecasting, transfer learning can potentially improve load prediction efficiency by allowing the model to learn from a diverse set of past forecasting tasks and adapt to new ones more quickly.

In this paper, we propose the use of transfer learning to solve the load forecasting problem. We begin by reviewing the existing literature on load forecasting and transfer learning, highlighting the challenges and limitations of traditional load forecasting approaches and the potential benefits of using transfer learning. Indeed, the core contribution of our work lies in the mathematical framework we propose, which addresses specific limitations of traditional load forecasting and transfer learning methods. Traditional approaches often lack a rigorous mechanism for selecting the most relevant pre-trained models for new tasks, leading to suboptimal performance. Our work fills this gap by introducing a novel task distance metric grounded in mathematical theory, which enables more precise and effective model selection. We then describe our proposed transfer learning-based approach for load forecasting, including a review of the task affinity score and its use in transfer learning. Finally, we present our experimental evaluation results, demonstrating the effectiveness of our approach.

Overall, this work’s contributions can be summarized as follows:

- In an empirical study, we demonstrate the usefulness of using the task affinity score as a measure of task distance for selection of the nearest source task from which to transfer knowledge (see Section 3.1).
- We propose a transfer learning approach integrating the task affinity score as a distance metric for source task selection (see Section 3.2).
- We improve the efficacy of load forecasting deep learning models in terms of training time and prediction score (see Section 4).

This paper is structured as follows. Section 2 comprehensively reviews the relevant literature related to transfer learning in load forecasting. Section 3 outlines the methodology used in this study, including the formulation of the task affinity score and the algorithm used in the experimental section. In Section 4, we present two case studies that illustrate the practical application of our methodology. Section 5 outlines future work that could build upon our findings and concludes the paper by summarizing our main findings and outlining their implications for future research and practice.

## 2. Literature Review

Accurate electric load forecasting is crucial for the safety and efficient operation of modern electric power systems, and various methods have been proposed to improve it. Several studies have suggested using transfer learning techniques to address the challenge of limited training data. For example, in [25], the authors proposed two deep learning models and a transfer learning framework to improve energy consumption prediction accuracy for buildings with limited data and demonstrated the effectiveness of the models through a case study of three office buildings. The proposed models, a sequence-to-sequence (seq2seq) model and a two-dimensional convolutional neural network with an attention layer, showed improved forecast accuracy over an extended memory network under a poor information state.

Similarly, in [26], the authors proposed a transfer learning-based artificial neural network model for one-hour-ahead building energy prediction to address the challenge of insufficient data for the training of data-driven predictive models for new buildings and existing buildings without advanced building automation systems. The study used data from 400 non-residential buildings from the open-source Building Genome Project to test the proposed method and found that transfer learning can effectively improve the accuracy of Back Propagation Neural Network (BPNN)-based building energy models for information-poor buildings with limited training data. The research also identified the most influential building features that influence the effectiveness of transfer learning, particularly in selecting appropriate source buildings and datasets.

In [27], Fang et al. proposed a novel hybrid deep transfer learning strategy to improve the accuracy of energy predictions in buildings with limited historical measurements. The approach combines long short-term memory and a domain adversarial neural network to extract temporal and domain-invariant features between source and target buildings. Experiments showed that this strategy significantly enhances building energy prediction performance compared to models trained on target or source-only data without transfer learning. The results can guide the effective use of existing building data resources. Another approach intended to be applied effectively to intelligent energy management in smart buildings was introduced in [28]. Using transfer learning and long short-term memory models, the authors proposed a new MEC-TLL framework for forecasting electric energy consumption in smart buildings. The framework uses a k-means clustering algorithm to group the daily load demand of many profiles in the training set. Then, it applies transfer learning to LSTM models to reduce computational time. The proposed approach was tested on two smart buildings in South Korea, and the results showed that it can reduce computational time while achieving superior performance compared to other models.

Zhou et al. proposed an integrated load forecasting model for an Integrated Energy System (IES) to improve energy scheduling [29]. The model addresses the problem of insufficient data for new users in the IES by combining Bidirectional Generative Adversarial Networks (BiGANs), data augmentation, and transfer learning techniques. The proposed model was compared to ten other data-driven models for two different types of users, namely residential and commercial, and found to be more accurate, on average, for each user type. The study also analyzed the impact of sample size, showing that the proposed model can improve the efficiency of other predictive models and can be used for load forecasting even when data are lacking. Peng et al. used a multi-source transfer learning-guided ensemble LSTM method (MTE-LSTM) to address the problem of insufficient energy data [30]. The process uses a two-stage source-domain building-matching method to find similar buildings and an LSTM modeling strategy that combines transfer learning and fine tuning to generate basic load forecasting models for the target building. An ensemble strategy is then used to weigh the output results of the basic forecasting models. The method was applied to multiple actual buildings and achieved high-precision load forecasting results when the target building data were relatively limited.

In another work [31], the authors found that a two-layer transfer learning-based architecture for short-term load forecasting (STLF) can improve the forecasting accuracy of load in a target zone. The architecture utilizes load data from source zones and includes an inner layer where latent parameters are introduced to represent the differences in electricity consumption behavior between zones. An iterative algorithm is developed in the outer layer to assign variant weights to datasets according to their fitness relative to the latent parameter-assisted model. Results from case studies showed that the proposed STLF architecture can improve the forecasting accuracy of classic STLF algorithms, mainly when the load data of the target zone are limited. Another work [32] presented a solution to the problem of developing predictive models for energy assets, such as electricity loads and PV power generation, using limited data. The authors proposed an energy-predictive model based on convolutional neural networks (CNNs) to capture time-series patterns, trends, and seasonalities in energy assets. They then proposed a transfer learning strategy to improve the model’s performance with limited training data. The approach was demonstrated in a case of daily electricity demand forecasting, and the results showed that the transfer learning strategy improves existing forecasting methods. The authors of [33] addressed the problem of insufficient data by training graph neural network (GNN)-based models in newly built residential neighborhoods. They proposed a transfer learning framework that uses knowledge from other areas with abundant data to assist the model in learning in areas with limited data. Specifically, the authors proposed an “attentive transfer framework” that ensembles GNN models trained from source domains and a GNN model trained on the target domain. The framework assigns dynamic weights to different GNN models based on the input data. The proposed framework was tested on real-world datasets, and the results showed that it is effective in various scenarios.

While existing research in load forecasting has explored various methodologies, including machine learning models and transfer learning techniques, these approaches often lack the adaptability needed for dynamic task environments. Most studies have focused on improving accuracy within a specific context, without addressing the broader challenge of model generalization across diverse tasks. Our work advances this field by introducing the ADTL framework, which uniquely incorporates a task distance metric to guide the selection of pre-trained models. Unlike traditional methods that apply a one-size-fits-all approach, our method dynamically adapts to the specific characteristics of each task, offering significant improvements in both accuracy and flexibility. This innovation positions our work as a critical contribution to the ongoing evolution of load forecasting methodologies.

## 3. Methodology

This section presents the task affinity score as a task-distance metric. We develop an empirical proof of the effectiveness of the TAS as the distance between two tasks. In this context, we define a task as a model–dataset pair for simplicity. For a source dataset ($X_{a}$), we train a model ($f_{a}$) using $L_{a}\left(\theta \right)$ as a loss function. The instigated distance is applied using a target dataset ($X_{b}$) and the same model ($f_{a}$) using the same loss function ($L_{a}\left(\theta \right)$).

### 3.1. Task Affinity Score

#### 3.1.1. Theoretical Formulation

The task affinity score is a measure based on Fisher information to approximate the similarity between two tasks. It determines how easily one task can gain knowledge from another task. It can help identify which tasks are most closely related and, therefore, most likely to benefit from shared knowledge [34].

First, we must define the Fisher information matrix to calculate the task affinity score. This matrix is a measure of the amount of information that is gained about a particular task after training. It is calculated by taking the expectation of the second derivative of the log likelihood of the loss function with respect to the task parameters. Once we define the Fisher information matrix, we use it to calculate the task affinity score. This is done by comparing the Fisher information matrices of the source and target tasks to determine the pseudo-distance between them. The greater the distance, the higher the task affinity score, indicating that knowledge gained from the source task is more likely to help learn the target task. For a neural network ($f_{\theta }$) with weights (θ) and a negative log-likelihood loss function (L(θ)), we define the Fisher information matrix as follows:

(1) $$\begin{array}{cc} F(\theta ) & =E\left[\nabla_{\theta }L\left(\theta \right)\nabla_{\theta }L{\left(\theta \right)}^{t}\right]\\ \; & =-E\left[H(L(\theta ))\right] \end{array}$$

where H is the Hessian matrix.

To calculate the Fisher information matrix in practice, we use an empirical approach, as shown in Equation (2).

(2) $$\hat{F}\left(\theta \right)=\frac{1}{\left|X\right|}\sum_{i\in X}\nabla_{\theta }L^{i}\left(\theta \right)\nabla_{\theta }L^{i}{\left(\theta \right)}^{t}$$

where for dataset X, $L^{i}\left(\theta \right)$ is the loss at the *i*th data point in the dataset.

The task affinity score between the source dataset ($X_{a}$) and the target dataset ($X_{b}$) is calculated using the Fréchet distance based on the Fisher information matrices of the network ($f_{\theta }$). $f_{\theta }$ is trained on the dataset ($X_{a}$). Specifically, the TAS is defined as follows:

(3) $$s\left[a,b\right]=\frac{1}{\sqrt{2}}Trace{\left(F_{a,a}+F_{a,b}-2{\left(F_{a,a}F_{a,b}\right)}^{\frac{1}{2}}\right)}^{\frac{1}{2}}$$

where $F_{a,a}$ is the Fisher information matrix of $f_{\theta }$ with source dataset $X_{a}$ and $F_{a,b}$ is the Fisher information matrix of $f_{\theta }$ with target dataset $X_{b}$.

The full Fisher information matrix is not used because it is computationally expensive to calculate in the ample space of neural network parameters. Instead, we calculate the diagonal approximation of the Fisher information matrix. These matrices are also normalized to have a unit trace. As a result, the TAS formula in Equation (3) can be simplified to the following form:

(4) $$\begin{array}{cc} s[a,b] & =\frac{1}{\sqrt{2}}∥F_{a,a}^{\frac{1}{2}}-F_{a,b}^{\frac{1}{2}}∥\\ \; & =\frac{1}{\sqrt{2}}{\left[\sum_{i}\left({\left(F_{a,a}^{ii}\right)}^{\frac{1}{2}}-{\left(F_{a,b}^{ii}\right)}^{\frac{1}{2}}\right)\right]}^{\frac{1}{2}} \end{array}$$

where $F_{a,a}^{ii}$ and $F_{a,b}^{ii}$ denote the diagonal elements of $F_{a,a}$ and $F_{a,b}$, respectively. The value of the TAS ranges from 0 to 1, where a score of 0 indicates a perfect similarity and a score of 1 indicates complete dissimilarity.

#### 3.1.2. Empirical Justification

**Figure 1:**
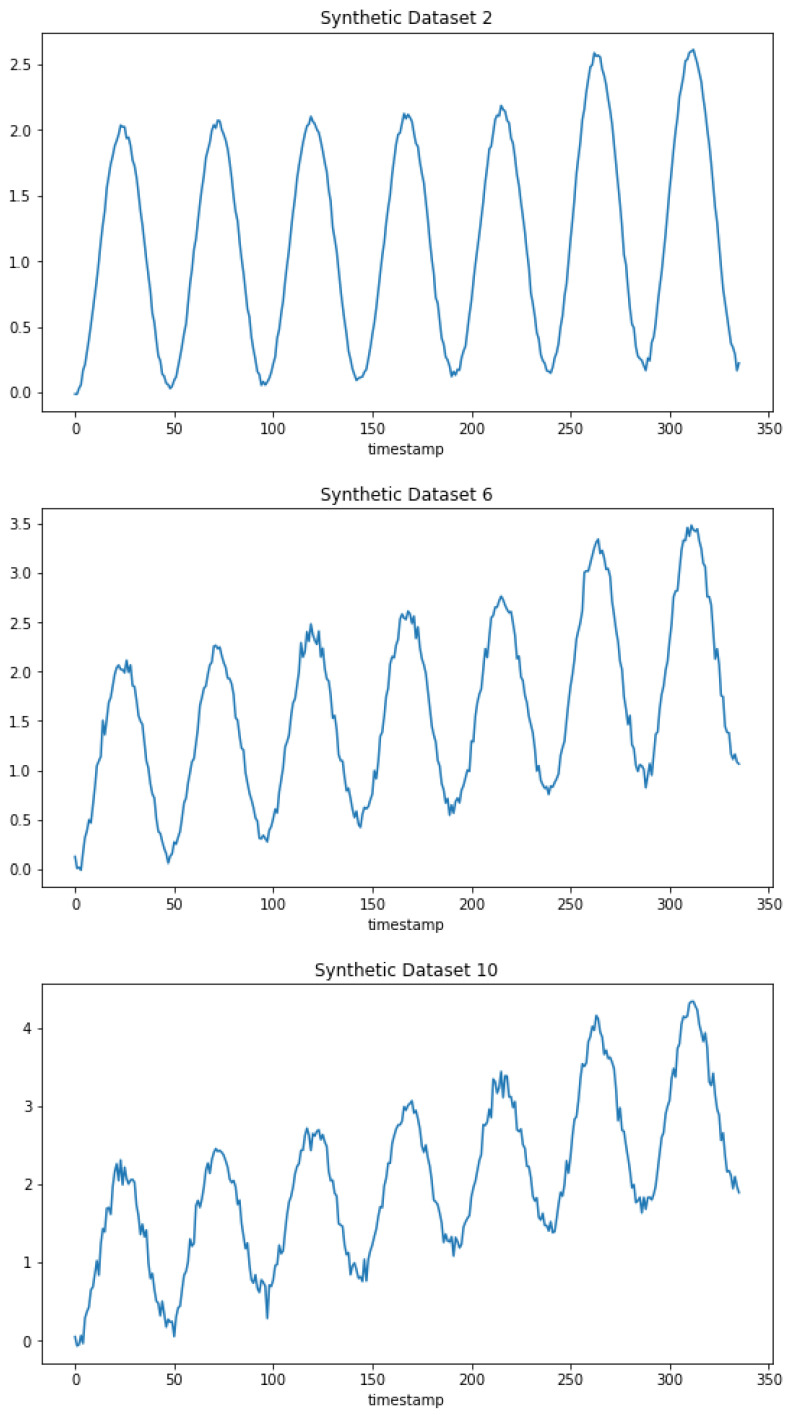
Synthetic datasets 2, 6, and 10.

We conducted a simulation using synthetic data to investigate the usefulness of the task affinity score as a measure of similarity between tasks. In this simulation, we added a linear trend and Gaussian noise information to a pseudo-sine function in a recursive manner to simulate the increasing difference between the datasets. The pseudo-sine function we used attempts to mimic the behavior of load data from week to week, as described in Equation (5). Figure 1 shows the difference between some datasets we used in this simulation.

(5) $$y_{i}=G\left(t\right).\left[1+\sin(2\pi t-\frac{\pi }{2})\right]+\tau_{i}\left(t\right)+\epsilon_{i}\left(t\right)$$

$$\begin{array}{cc} G\left(t\right)=1_{weekdays}\left(t\right)+\alpha .1_{weekdays}\left(t\right) & \;\alpha =1.7\\ \tau_{i}\left(t\right)=k_{i}t & \;k_{i}\in \left[0,1\right]\\ \epsilon \left(t\right)∼N\left(0,\;\sigma_{i}^{2}\right) & \;\sigma_{i}\in \left[0.1,1\right] \end{array}$$

where *t*, $k_{i}$, and $sigma_{i}$ are linearly spread in the respective intervals. We used 48 data points to simulate one day (although it is not necessary, we attempted to mimic the 30 min sampling rate used in the datasets in the experimental section). We used ten datasets.

**Figure 2:**
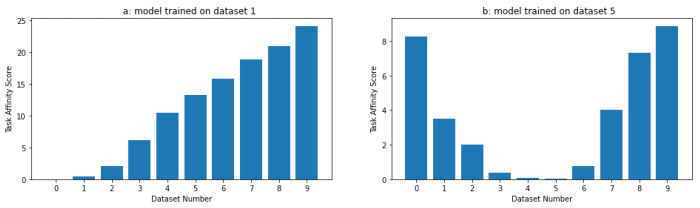
Task affinity scores between the different data and tasks 0 and 5.

Our results showed that as the difference between the datasets increased, the task affinity score consistently increased, indicating that the task affinity score effectively captured the increasing dissimilarity between the tasks. This is clear from Figure 2, where we trained two models for a few epochs on dataset 1 and dataset 5. The task affinity score increased as we strayed further away from the primary dataset. This simulation supports the intuition behind using the task affinity score as a measure of similarity between tasks, as it demonstrates that the metric is sensitive to changes in the differences between the datasets. Overall, our simulation results support the use of the task affinity score as a reliable and valid measure of the dissimilarity between tasks.

#### 3.1.3. TAS vs. MSE

This section compares the task affinity score (TAS) and the mean squared error (MSE) as metrics to measure the distance between tasks. We trained a fully connected neural network and a long short-term memory (LSTM) model on the same datasets from the previous experiment (first and sixth datasets). We compared the distances between all tasks to these target datasets. To evaluate the performance of the TAS and MSE metrics, we computed the distance between every task (trained models and respective datasets) and the target task using both metrics and compared the results.

**Figure 3:**
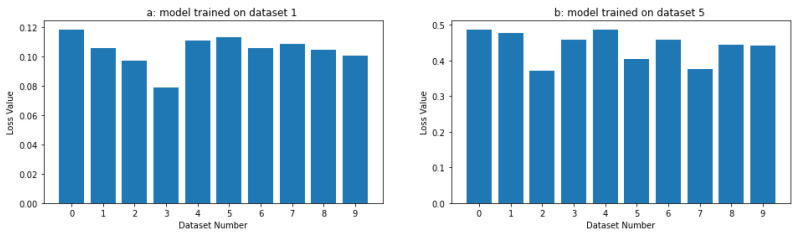
Task affinity score between the different datasets on tasks 0 and 5.

The results in Figure 3 show that the TAS metric outperforms the MSE loss in selecting the appropriate initial task to transfer the knowledge. Comparing Figure 2 and Figure 3, we can see how the loss function does not show the same pattern as the TAS distance. In particular, the TAS was more reliable at identifying the nearest tasks and distinguishing dissimilar tasks, allowing the model to converge faster while requiring fewer training epochs. In contrast, the MSE loss was inconsistent, leading to less efficient model selection. On the other hand, we trained different fully connected neural networks and LSTM models with random weights. We calculated the number of epochs required to achieve the same performance as the nearest (and the second nearest) neural network. We present the results in Table 1.

In conclusion, our empirical study demonstrates that the TAS metric is a superior choice for measuring the distance between tasks compared to the MSE loss. The TAS metric is more accurate and consistent in identifying similar tasks and distinguishing dissimilar tasks.

### 3.2. Affinity-Driven Transfer Learning

**Figure 4:**
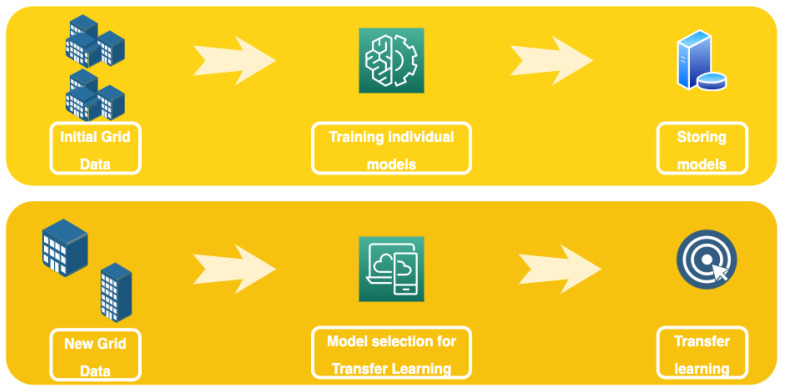
Affinity-driven transfer learning diagram.

This paragraph presents the methodology behind the affinity-driven transfer learning algorithm. As shown in Figure 4 and detailed in Algorithm 1, the algorithm has two steps, namely a learning step and a transfer learning step. In the first step, we train different algorithms on different elements of a particular grid. The elements are usually historical household electricity data. The models are then stored for future use. The second step is selecting a suitable model to transfer knowledge, from which we add new elements to the grid by calculating the nearest task in terms of the task affinity score.
**Algorithm 1** Affinity-Driven Transfer Learning**Input:** Old grid elements Electricity Demand (OGTS) **Input:** New grid element Electricity demand (NGTS) **Output:** $h^{*}$  ***I - pretraining:*** 1: train $h_{i}$ models on ∀i∈{1…m} dataset from OGTS  ***II - Transfer Learning:*** 2: $h^{*}=\min_{h_{i},i\in \left\{1\ldots m\right\}}\;TAS\left(\tau_{i},tau_{new}\right)$ 3: Train $h^{*}$ for few epochs 4: **return** $h^{*}$

### 3.3. Models and Metrics

#### 3.3.1. Fully Connected Neural Networks

A fully connected network, also known as a fully connected layer or a dense layer, is an artificial neural network in which each neuron in a layer is connected to every neuron in the previous layer. In other words, each neuron in a fully connected layer receives input from all the neurons in the previous layer [35].

Let us consider a fully connected layer with *n* inputs and *m* outputs [36], where the inputs are represented by a vector x of size *n* and the outputs are represented by a vector y of size *m*. Each output ($y_{i}$) is computed as a weighted sum of the inputs ($x_{j}$) plus a bias term ($b_{i}$), then passed through an activation function (*g*) as shown in Equation (6).

(6) $$y_{i}=g\left(\sum_{j=1}^{n}w_{ij}x_{j}+b_{i}\right)$$

where $w_{ij}$ represents the weight of the connection between the *j*th input and the *i*th output and $b_{i}$ represents the bias term for the *i*th output.

This equation can also be represented in matrix form as follows:

y=σ(Wx+b)

where W is a weight matrix of size (m×n), b is a bias vector of size *m*, x is the input vector of size *n*, and σ is the sigmoid function that operates on the elements of the output vector (y).

In a multilayer neural network, the output of one fully connected layer is typically fed as input to the next fully connected layer, and so on, until the final output layer is reached.

#### 3.3.2. LSTMs

LSTM models are gated recurrent neural networks [37] used in various areas, such as image generation [38], speech recognition [39], natural language processing [40], and time-series forecasting [41].

**Figure 5:**
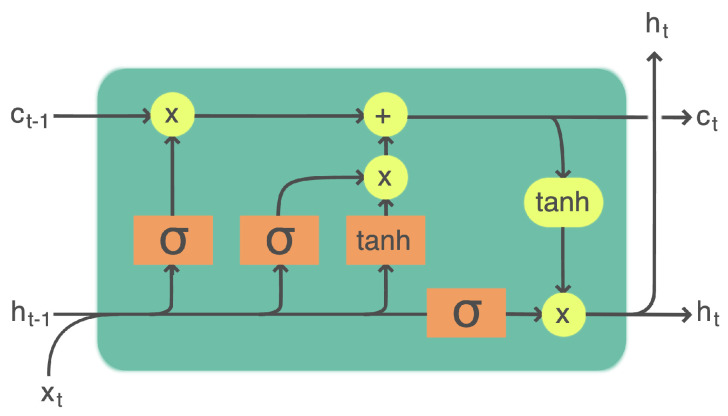
A long short-term memory cell.

An LSTM model uses the same weights for every timestamp. It takes the input sequence element by element and carries hidden information from one timestamp to the next, as shown in Figure 5. The classic recurrent neural network (RNN) fails to carry information from long in the past because of the gradient vanishing problem. On the other hand, an LSTM model uses gates to carry different information when scanning the input sequence. So, instead of one simple activation function, an LSTM model uses Equations (7)–(12) as follows:

(7) $$\begin{array}{cc} i_{t} & =\sigma (W_{i}\cdot h_{t-1}+U_{i}\cdot x_{t}+P_{i}\cdot C_{t-1}+b_{i}) \end{array}$$

(8) $$\begin{array}{cc} f_{t} & =\sigma (W_{f}\cdot h_{t-1}+U_{f}\cdot x_{t}+P_{f}\cdot C_{t-1}+b_{f}) \end{array}$$

(9) $$\begin{array}{cc} {\tilde{C}}_{t} & =\psi (W_{c}\cdot h_{t-1}+U_{c}\cdot x_{t}+b_{c}) \end{array}$$

(10) $$\begin{array}{cc} C_{t} & =f_{t}⊙C_{t-1}+i_{t}⊙{\tilde{C}}_{t} \end{array}$$

(11) $$\begin{array}{cc} o_{t} & =\sigma (W_{o}\cdot h_{t-1}+U_{o}\cdot x_{t}+P_{o}\cdot C_{t-1}+b_{o}) \end{array}$$

(12) $$\begin{array}{cc} h_{t} & =o_{t}⊙\psi \left(C_{t}\right) \end{array}$$

where subscripts *i*, *f*, and *o* denote the input, forget, and output gates, respectively; *h* denotes the hidden state vector; *C* is the long-term state vector; $W_{i}$, $W_{f}$, $W_{o}$, and $W_{c}$ represent the weight matrices of the hidden information from the last timestamp; $U_{i}$, $U_{f}$, $U_{o}$, and $U_{c}$ represent the weight matrices of the input information; and $P_{i}$, $P_{f}$, $P_{o}$, and $P_{c}$ represent the weight matrices of the long-term state (*C*). The terms $b_{i}$, $b_{f}$, and $b_{o}$ are biases of the gates. σ is the sigmoid operator, ψ is the hyperbolic tangent function (tanh), and ⊙ is the element-wise multiplication operator.

#### 3.3.3. Metrics

To evaluate the accuracy of our proposed model, we relied on commonly used evaluation metrics, namely the Root Mean Square Error (RMSE) and the Mean Absolute Percentage Error (MAPE), as represented in Equations (13) and (14), respectively.

(13) $$RMSE\left(y,\hat{y}\right)=\sqrt{\frac{\sum_{t=1}^{n}{\left|y_{t}-{\hat{y}}_{t}\right|}^{2}}{n}}$$

(14) $$MAPE\left(y,\hat{y}\right)=\frac{1}{n}\sum_{t=1}^{n}\left|\frac{y_{t}-{\hat{y}}_{t}}{y_{t}}\right|$$

## 4. Case Study

In this experimental section, we present two case studies that compare the AEMO dataset’s forecasting performance using the ADTL algorithm and an initialized network. In the second case study, we extend our analysis to investigate the performance of the nearest model and random network over different forecast horizons. By doing so, we aim to identify whether the relative performance of each model remains consistent across various time horizons. The selected datasets are highly relevant to electricity consumption, where transfer learning significantly enhances predictive accuracy and model efficiency. By focusing on open-source datasets, we ensure that our results are replicable and accessible to the broader research community, fostering transparency and further innovation. Additionally, the datasets are relatively clean, allowing us to concentrate on the core contributions of our paper rather than data preprocessing challenges, thereby providing a more explicit demonstration of the effectiveness of our proposed methods.

### 4.1. Case Study 1: Australian Dataset

The Australian Energy Market Operator (AEMO) dataset [42] is a collection of energy market data from the National Electricity Market (NEM) in Australia. The NEM is a wholesale electricity market that covers the eastern and southeastern parts of Australia. The AEMO dataset includes data on electricity demand and supply in the NEM. These data are collected in real time from market participants, including electricity generators, retailers, and transmission network service providers, and are used to manage the operation of the electricity grid and ensure a reliable supply of electricity to consumers.

In this work, we selected a few datasets from different parts of the market. We covered different years and locations to obtain the spatial and temporal differences between the datasets. The time series were selected from Queensland, New South Wales, Victoria, South Australia, and Tasmania, as detailed in Table 2.

In this study, we split our datasets into a meta subset and a query subset. The meta subset was used to train multiple models, and the query subset was used to evaluate their transferability. Specifically, we trained five different models on the five datasets from the meta subset and evaluated their performance when used on the datasets from the query subset.

**Figure 6:**
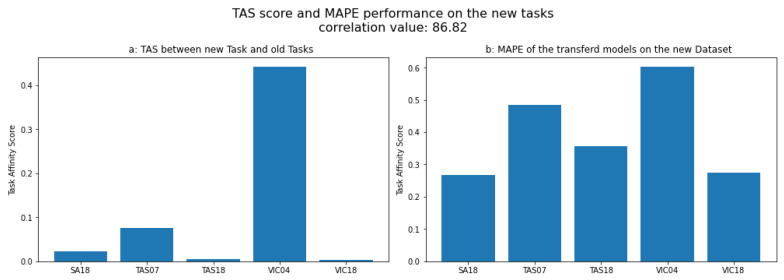
Average performance on different query sets.

To evaluate the transferability of knowledge from the meta tasks to new tasks, we calculated the task affinity score distance between the meta tasks and each of the query tasks. We then measured the average performance after transferring knowledge from the meta models to each task from the query set. We found a high correlation between the MAPE performance and the task affinity score distance. Figure 6 shows two subfigures labeled Figure 6a and Figure 6b. Figure 6a illustrates the task affinity score (TAS) applied to each dataset from the meta set with respect to the QLD18 dataset.

On the other hand, Figure 6b displays each model’s mean absolute percentage error performance from the meta set trained on the QLD18 dataset from the query dataset. This figure shows a very high correlation between the performance of the models and the distance between tasks calculated by the TAS. We list the different correlations of the same experiment with different datasets from the query set in Table 3. The correlation between the proposed TAS distance and the MAPE performance is consistently high, with a mean value of 88.78%. In this experiment, to ensure the stability of the TAS metric, we experimented with each of the query sets ten times and averaged the results. These results indicate that the similarity between the meta tasks and the query tasks influences the transferability of knowledge from the meta models to new models. In particular, the closer the task affinity score between the two sets of tasks, the better the transferability of knowledge.

In addition to the results discussed above, we trained the nearest and the second nearest models to further compare the performance using the TAS information and trained the model from scratch. In Table 4, we list the MAPE of the models that were pre-trained on the different datasets of the meta set on the query datasets. We performed the experiment for zero, one, and five epochs. The results indicate that the knowledge gained from the nearest task made the models faster in terms of epoch convergence. The results from the second nearest task indicate that transferring the knowledge even from the second nearest task is still a better starting point for training neural networks than training them with random weights. The average MAPE of the nearest model is 0.29 without training, compared to an average of 0.95 when we trained a random FCN for five epochs.

**Figure 7:**
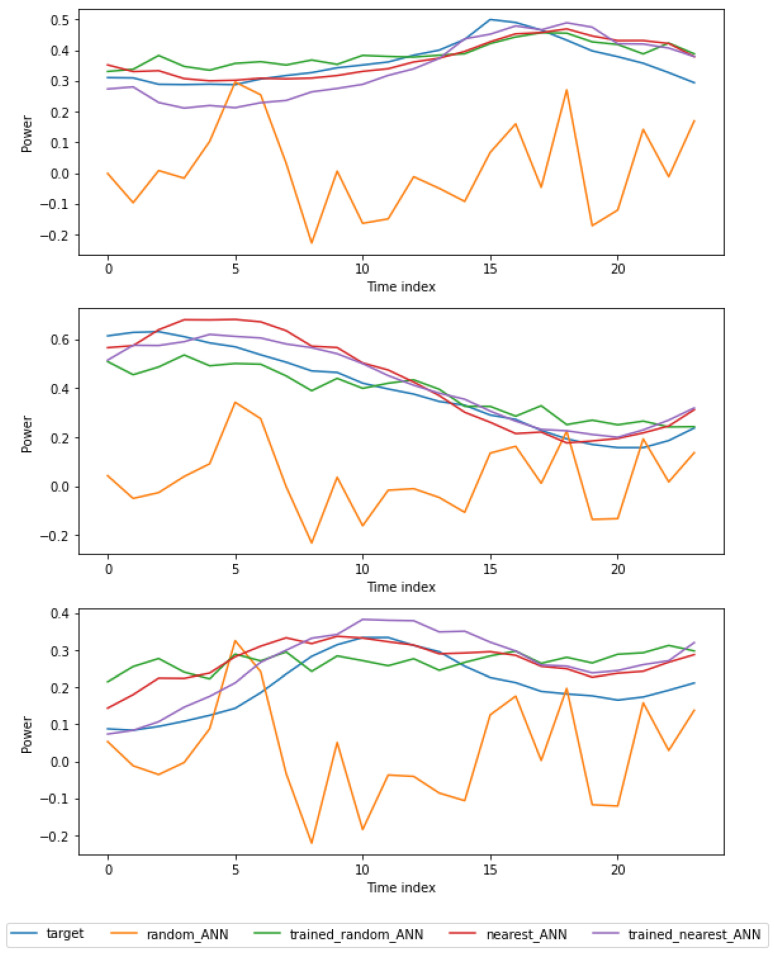
Forecasting samples at different learning levels.

Furthermore, we downsampled the datasets from the query set to 10%, 20%, and 50% of their original sizes. Downsampling simulates data scarcity in this context. Table 5 shows the MAPE performance of the nearest and second nearest models when trained on different datasets. The results demonstrate that even with a reduction in data due to downsampling, transfer learning from the nearest dataset can result in good performance. In all cases, the nearest model performed well compared to the models trained on the whole dataset, indicating that the knowledge transferred from the nearest task overcame the data scarcity. For example, the average MAPE performance was better by a factor of 32% when using the pre-trained model and a random FCN. In Figure 7, we show the performances of different networks in the experiment. The figure clearly shows how the nearest model achieves better forecast ability than the random network.

### 4.2. Case Study 2: Smart Grid Dataset

“The Smart-Grid Smart-City Customer Trial Data” dataset was collected as part of a trial conducted by the Australian Government Department of Climate Change, Energy, the Environment, and Water [43]. The dataset contains electricity consumption data from around 1300 households in New South Wales, Australia, collected over a period of 12 months. The data include half-hourly electricity consumption readings.

In the context of transfer learning for load forecasting in an electricity grid, the meta set can be seen as a set of pre-trained models that have already learned to forecast the load for some subset of elements in the grid. These models were trained on historical data and can be thought of as “experts” in predicting the load for those specific elements. The query set, in this case, refers to the new models that need to be trained for forecasting of load for previously unseen or new elements in the same electricity grid. These new models need to be trained on a smaller amount of data as compared to the pre-trained models in the meta set. The goal of transfer learning in this scenario is to leverage the knowledge learned by the pre-trained models on the elements they have already forecasted to improve the accuracy and efficiency of training of the new models for the new elements.

The primary goal of our experiments on this dataset was to evaluate the effectiveness of our approach in transferring knowledge from a set of known source apartments to forecast the electricity consumption in a target apartment. Specifically, we sought to determine whether the TAS could identify the most relevant source task for a given target task and whether this approach could speed up the transfer learning process. We selected a sample of 20 random apartments as the meta set and a sample of 30 apartments as the query set. Then, we trained 20 different LSTM models on the apartments’ data from the meta set, serving as the different possible sources for the query set of apartment data.

To evaluate the performance of ADTL, we used the mean absolute percentage error (MAPE) and the root mean square error (RMSE). Overall, our experiments on the dataset demonstrate the potential of ADTL as an effective transfer learning approach when the source and target tasks are closely related. In Table 6, we present a comparison between a random LSTM model and the selected LSTM model using the ADTL approach. We evaluated the algorithm’s performance by comparing the results of a randomly initialized LSTM model with the results of the nearest LSTM model in terms of task distance. After training for five epochs, the random network’s performance could not be improved and remained very poor for all prediction windows and horizons (1 day, 3 days, and 7 days), with an average mean absolute percentage error (MAPE) of 1.83 and an average root mean squared error (RMSE) of 0.233. In contrast, the nearest LSTM model showed significant improvement in performance after being trained for five epochs, achieving high accuracy for all prediction horizons, with an average MAPE and of of 0.73 and an average RMSE of 0.196. These results highlight the limitations of traditional deep learning models in learning from new and unseen data and demonstrate the potential of our transfer learning approach to improve their performance.

As shown in Table 7, we conducted experiments with reduced data samples from each apartment to simulate data scarcity issues. We downsampled the data from the query set. We present the average performance of a randomly generated LSTM network and the nearest LSTM network in terms of TAS. Our results demonstrate that the nearest LSTM network outperformed the randomly generated network. Notably, after only five epochs of training, the nearest LSTM network achieved performance comparable that achieved following training on the complete dataset without downsampling.

## 5. Future Work and Conclusions

Our results suggest that the TAS metric should be given more consideration as a metric for evaluating the transferability of machine learning models in real-world applications, particularly in scenarios where efficiency and convergence speed are important considerations. This finding has important implications for the design of meta-learning algorithms and suggests that incorporating task similarity information can improve the performance of these algorithms. In particular, MAML [44] was used as a part of the transferable model-agnostic meta-learning (T-MAML) approach [45], proposing an approach for load forecasting for single households that enables multiple households to collaboratively train a generic artificial neural network (ANN) model, then further train the model at each target household node for the purpose of STLF. We propose the use of the TAS distance to select the subset of the dataset used in the meta-learning phase to minimize the number of learning steps instead of a random selection.

In this paper, we present empirical evidence to support the use of the task affinity score (TAS) as a reliable and effective distance measure for task similarity in transfer learning. Our study also introduces a new transfer learning algorithm called affinity-driven transfer learning (ADTL), which leverages the TAS to select the most appropriate source task for knowledge transfer. To evaluate the effectiveness of ADTL, we conducted experiments on two datasets, namely the AEMO dataset and the smart apartment dataset. Our results demonstrate that ADTL outperformed traditional transfer learning approaches in terms of mean absolute percentage error (MAPE) across both datasets. These findings suggest that ADTL can successfully identify the most relevant source task for knowledge transfer based on the task affinity score. Furthermore, we investigated the effectiveness of ADTL under data scarcity conditions. To simulate this scenario, we downsampled the datasets to 10%, 20%, and 50% of their original sizes. Our experiments showed that even under these data-scarce conditions, ADTL continued to perform significantly better than randomly initialized models. This demonstrates the potential of ADTL to address the data scarcity problem in transfer learning and suggests that it is a promising approach for real-world applications. Overall, our study provides important insights into the use of thee TAS as a distance measure for task similarity in transfer learning and highlights the potential of ADTL as an effective transfer learning approach. Future research in this area could further refine the use of the TAS in transfer learning and explore additional approaches to leverage task similarity to improve knowledge transfer. 

## Figures and Tables

**Table 1:** Number of epochs an LSTM (a fully connected ANN) needs to match the MAPE performance of a one-epoch pre-trained model of the nearest dataset and the second nearest dataset.

Source	Nearest Dataset Performance		Second Nearest Dataset Performance	
	LSTM	FCN	LSTM	FCN
dataset 0	7	3	7	5
dataset 1	7	3	7	5
dataset 2	7	3	8	4
dataset 3	8	6	5	5
dataset 4	5	4	5	3
dataset 5	6	3	6	5
dataset 6	8	3	7	4
dataset 7	8	3	7	4
dataset 8	5	5	7	6
dataset 9	7	4	7	6

**Table 2:** Dataset locations and years used in experiment 1.

Dataset Name	Location	Year
NSW03	New South Wales	2003
NSW18	New South Wales	2018
QLD03	Queensland	2003
QLD18	Queensland	2018
SA03	South Australia	2003
SA18	South Australia	2018
TAS07	Tasmania	2007
TAS18	Tasmania	2018
VIC04	Victoria	2004
VIC18	Victoria	2018

**Table 3:** Correlations between the TAS distance and the MAPE on the query datasets.

Dataset Name	Correlation Value
NSW03	89.10
NSW18	88.45
QLD03	86.89
QLD18	86.82
SA03	87.94

**Table 4:** MAPE performance of the selected pre-trained neural network in comparison with a random neural network.

Source	Nearest Task			Second Nearest Task			Random FCN		
Number of Epochs	0	1	5	0	1	5	0	1	5
NSW03	0.33	0.27	0.14	0.55	0.49	0.41	0.98	0.92	0.72
NSW18	0.23	0.22	0.15	0.54	0.47	0.32	1.18	1.08	0.85
QLD03	0.37	0.29	0.24	0.63	0.58	0.37	1.78	1.40	1.22
QLD18	0.26	0.21	0.20	0.62	0.49	0.46	1.33	1.29	0.94
SA03	0.29	0.20	0.18	0.50	0.48	0.43	1.54	1.49	1.02

**Table 5:** Performance of the selected pre-trained neural network and a random neural network on downsampled datasets.

Source	Nearest Task Neural Network Performance (MAPE)			Average of Random Neural Networks Performances (MAPE)		
	10%	20%	50%	10%	20%	50%
NSW03	0.28	0.28	0.27	0.65	0.61	0.58
NSW18	0.34	0.33	0.24	0.52	0.52	0.48
QLD03	0.51	0.46	0.40	0.44	0.43	0.41
QLD18	0.37	0.34	0.27	0.69	0.63	0.59
SA03	0.48	0.47	0.29	0.68	0.57	0.53

**Table 6:** Performance of the selected pre-trained neural network in comparison with a random neural network.

	1-Day Data		3-Day Data		7-Day Data	
	MAPE	RMSE	MAPE	RMSE	MAPE	RMSE
Random LSTM	2.25	0.340	1.98	0.313	2.25	0.349
Random LSTM (trained for 5 epochs)	1.75	0.211	1.72	0.237	2.01	0.251
Nearest LSTM	0.81	0.201	0.77	0.199	0.85	0.208
Nearest LSTM (trained for 5 epochs)	0.73	0.195	0.72	0.195	0.75	0.198

**Table 7:** Performance of the selected pre-trained neural network and a random neural network on downsampled datasets.

	10% Downsampling		20% Downsampling		50% Downsampling	
	MAPE	RMSE	MAPE	RMSE	MAPE	RMSE
Random LSTM	3.73	0.537	3.35	0.552	3.64	0.532
Random LSTM (trained for 5 epochs)	3.58	0.530	3.11	0.415	3.51	0.304
Nearest LSTM	0.92	0.238	0.83	0.225	0.79	0.182
Nearest LSTM (trained for 5 epochs)	0.82	0.199	0.80	0.202	0.75	0.197

## Data Availability

The data used in this study are available online at https://aemo.com.au/energy-systems/electricity/524national-electricity-market-nem/data-nem/aggregated-data (accessed on 16 August 2024) and https://data.gov.au/dataset/ds-dga-4e21dea3-9b87-4610-94c7-15a8a77907ef/details?q=smart20grid20smart20527city (accessed on 16 August 2024).
